# Axial length change and its relationship with baseline choroidal thickness – a five-year longitudinal study in Danish adolescents: the CCC2000 eye study

**DOI:** 10.1186/s12886-020-01427-8

**Published:** 2020-04-15

**Authors:** Mathias Hvidtfelt Hansen, Line Kessel, Xiao Qiang Li, Anne Mette Skovgaard, Michael Larsen, Inger Christine Munch

**Affiliations:** 1 Department of Ophthalmology, Rigshospitalet, Valdemar Hansens Vej 1-23, afsnit 37, 2600 Glostrup, Denmark; 2 Department of Clinical Medicine, Faculty of Health Sciences, University of Copenhagen, Copenhagen, Denmark; 3 National Institute of Public Health, University of Southern Denmark, Copenhagen, Denmark; 4 Department of Ophthalmology, Zealand University Hospital, Roskilde, Denmark

**Keywords:** Cohort study, Children, Choroidal thickness, Axial length, Incident myopia, CCC2000

## Abstract

**Background:**

Myopic eyes are longer than nonmyopic eyes and have thinner choroids. The purpose of present study was to investigate whether a thinner subfoveal choroid at 11 years of age predicted axial eye elongation and myopia during adolescence.

**Methods:**

Longitudinal, population-based observational study. Axial length was measured using an interferometric device and choroidal thickness was measured by spectral-domain optical coherence tomography. Myopia was defined as non-cycloplegic subjective spherical equivalent refraction ≤ − 0.50 diopters.

**Results:**

Right eyes of 714 children (317 boys) were examined at age (median (IQR)) 11.5 (0.6) years and 16.6 (0.3) years during which axial length (median (IQR)) increased by 243 (202) μm in eyes without myopia (*n* = 630) at baseline compared with 454 (549) μm in eyes with myopia (*n* = 84) at baseline, *p* < 0.0001. A thicker baseline subfoveal choroid was associated with increased five-year axial elongation after adjustment for baseline axial length in nonmyopic eyes (β = 27 μm/100 μm, 95%CI 6 to 48, *p* = 0.011) but not in myopic eyes (*p* = 0.34). Subfoveal choroidal thickness at 11 years of age did not predict incident myopia at 16 years of age (*p* = 0.11). Longer baseline axial length was associated with greater five-year axial elongation in both myopic (β = 196 μm/mm, 95%CI 127 to 265, *p* < 0.0001) and nonmyopic eyes (β = 28 μm/mm, 95%CI 7 to 49, *p* = 0.0085) and the odds for incident myopia increased with 1.57 (95%CI 1.18 to 2.09, *p* = 0.0020) per mm longer axial length at baseline.

**Conclusion:**

A thin subfoveal choroid at age 11 years did not predict axial eye elongation and incident myopia from age 11 to 16 years. A longer eye at age 11 years was associated with greater subsequent axial eye elongation and with increased risk of incident myopia at age 16 years.

## Background

Myopia is predominantly caused by abnormal axial elongation of the eye [1]. In emmetropic eyes growth is believed to be guided by visual inputs to the retina with hyperopic inputs stimulating axial elongation and myopic inputs inhibiting it. This association has been found in a number of species such as chicks [2, 3], rhesus monkeys [4], marmosets [5–7] and humans [8]. Experimental settings in both animals [9–12] and humans [8] show a modulation of choroidal thickness in response to visual inputs which also leads to choroidal secretion of scleral growth regulators [13]. Consequently, the choroid is believed to play an active part in eye growth regulation [14, 15]. Eyes that develop myopia both experience a thinning of the choroid and an elongation of the eyeball [16, 17] and a study following 101 Australian children over an 18 month period found an association between choroidal thinning and increasing axial length growth [18]. Whether choroidal thinning precedes or follows axial elongation is, however, unknown. The main aim of present study was therefore to investigate whether a thinner choroid at 11 years of age predicted subsequent five-year axial eye elongation and incident myopia.

## Methods

### Study population

**Fig. 1:**
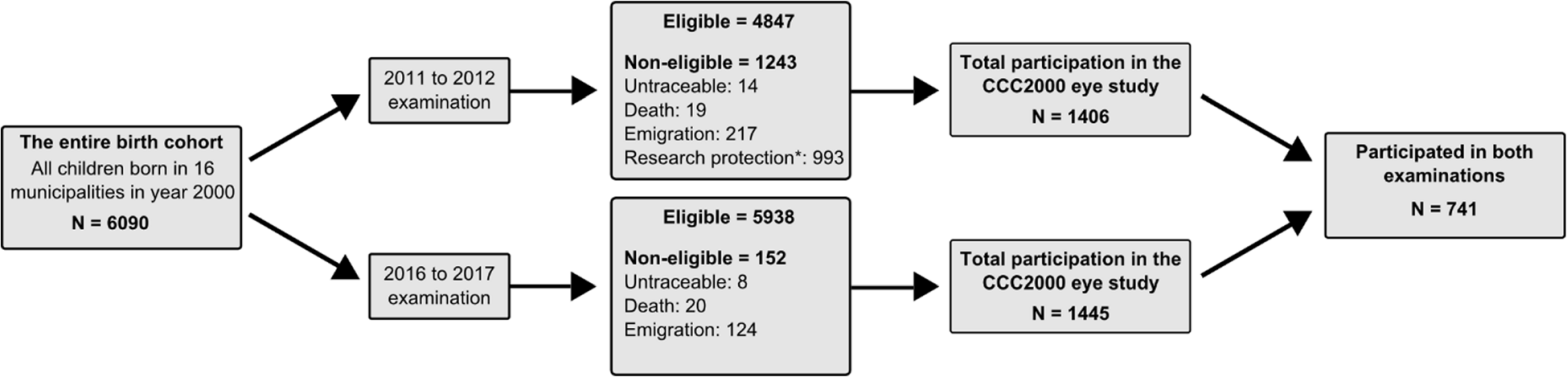
Ascertainment of participants. Research protection (*) refers to a procedure in force until April 1, 2014 whereby citizens could opt for a general exemption from being invited to participate in research projects through the national civil registry

The Copenhagen Child Cohort 2000 eye study is a prospective, population-based, observational study of children born in the year 2000 in 16 municipalities of Copenhagen County, Denmark. The Copenhagen Child Cohort 2000 (CCC2000) includes 6090 children and was initiated with the main objective to study mental health and development from birth to adulthood [19]. The cohort is representative of the Danish population regarding basic child-related parameters [20]. The first eye examination was performed in year 2011 to 2012 and included 1406 participants [21]. Between August 20, 2016 and August 31, 2017, all eligible children of the cohort were invited to a new examination of which 1445 volunteered to participate. A total of 741 subjects participated in the eye examinations in both 2011–2012 and 2016–2017 (Fig. 1). A higher proportion of the girls (412/733, 56%) than the boys (329/673, 49%) from the baseline examination in 2011–2012 participated in the follow-up examination in 2016–2017. There were no differences in body height, axial length, choroidal thickness, spherical equivalent refraction or birth-related parameters (*p* > 0.30) between the children who attended both examinations and the children who only participated in the 11 to 12-year examination. Medical history was obtained from the participants or their parents/legal guardians.

We excluded a total of 27 subjects. Among them, eight were due to a best corrected visual acuity < 80 Early Treatment Diabetic Retinopathy Study (ETDRS) letters at age 16, 15 due to missing subjective refraction at age 11–12 years, two due to missing axial length measurements at age 11–12 years, and two due to missing or poor quality optical coherence tomography (OCT) scans. A total of 714 participants and their right eyes were included in the analyses.

### Procedures

Eye examinations included best corrected visual acuity (BCVA), measured using ETDRS charts (4-m original series; precision-Vision, La Salle, IL, USA) as the maximal number of letters the children could read with optimal correction. Optimal correction was determined by non-cycloplegic subjective refraction guided by an autorefractor. The Retinomax K-plus (Right MFG Co., Ltd., Tokyo, Japan) was used in 2011 and the Nidek AR-660A (NIDEK CO., LTD., Gamagori, Aichi 443–0038, Japan) in 2016. The refraction suggested by the autorefractor was used in the following subjective measurement of refraction. When the autorefractor measured a negative spherical refraction, we used a 0.5 D less negative correction than suggested. Wearing this starting correction, the children were asked to read as many letters on an ETDRS chart as possible. Hereafter, positive lens power (+ 0.5 D) was added continuously until a significant blur occurred followed by the adding of − 0.25 D lenses until there was no longer a gain in letters read. We calculated the spherical equivalent refraction by adding half of the negative cylinder refraction to the spherical refraction. Eyes with a spherical equivalent refraction ≤ − 0.50 D were defined as myopic.

Axial length measurements were performed using a partial coherence interferometric device (IOL-Master, version 3.01.0294; Carl Zeiss Meditec, La Jolla, CA, USA) based on an average of at least 5 scans. The device measured axial length as the distance between cornea and retinal pigmented epithelium and thereafter automatically adjusted for the distance between pigment epithelium and inner limiting membrane. The displayed axial length values were thus the adjusted values corresponding to the distance between cornea and inner limiting membrane.

Five-year change in axial length was defined as the difference between axial length at age 11 and 16 years, divided by the follow-up time in years and then multiplied by five.

The macular choroid was imaged using enhanced depth imaging spectral domain optical coherence tomography (EDI-SD-OCT; Spectralis HRA + OCT; Heidelberg Engineering, Heidelberg, Germany) with two scan protocols: a horizontal scan including seven scan lines within a 5° × 30° rectangle and a 4-line 30° radial scan. Each scan line consisted of an average of 25 B-scans. The horizontal scan line with the deepest foveal depression and the presence of a pronounced foveal center specular reflex was used in the evaluation of subfoveal choroidal thickness. Choroidal thickness was measured using the manufacturer’s software (Heidelberg Eye Explorer, version 1.6.1.0; Heidelberg Engineering) by manually moving the segmentation line depicting the inner limiting membrane to the choroidoscleral border. All measurements of choroidal thickness were performed by the same operator (XQL). Intragrader variability has been assessed before, showing an intraclass correlation coefficient of 0.996 [22]. We used the subfoveal choroidal thickness in all analyses which would be referred to as just choroidal thickness.

Participants’ height was measured to the nearest 0.1 cm using a wall-mounted altimeter (Height Measuring Rod 5002.02; Soehnle Professional GmbH & Co., Backnang, Germany) and body weight using an electronic scale (Exact/personal scale 6295; OBH Nordica Denmark A/S, Taastrup, Denmark) to the nearest 0.1 kg.

### Statistics

The SAS® Enterprise Guide statistical software package (version 7.2; SAS Institute, Cary, NC, USA) was used for all statistical analyses. Normally distributed data were reported as mean ± standard deviation (SD) and non-normally distributed data as median ± interquartile range (IQR). The effects of baseline choroidal thickness, axial length, body height, age and sex on the five-year change in axial length were assessed in crude analyses using Student’s *t*-tests and linear regression. We performed a multivariate regression analysis including both baseline axial length and choroidal thickness and a multivariate analysis in a general linear model that included, baseline choroidal thickness, axial length, body height, age and sex. The odds ratios (OR) for incident myopia were calculated using logistic regression models and adjusted as aforementioned. We used the Pearson correlation coefficient to evaluate the correlation between subfoveal choroidal thickness and axial length. Body mass index was calculated as body weight in kilograms divided by the square of the body height in meters. We tested all variables for interaction with sex and baseline myopia.

## Results

We analyzed the right eyes of 714 (317 boys, 397 girls) participants with a median (IQR) age at follow-up of 16.6 (0.3) years and a median (IQR) follow-up time of 5.1 (0.5) years (Table 1). The median (IQR) 5-year increase in axial length from age 11 to 16 years was 248 (225) μm (Table 1).

**Table 1:** Characteristics of the 714 study participants and their right eyes at the baseline and follow-up examination

Parameter	Baseline, year 2011–12	Follow-up, year 2016–17
Age, median (IQR), years	11.5 (0.6)	16.6 (0.3)
Follow-up time, median (IQR), years	–	5.1 (0.5)
Best corrected visual acuity, mean (SD), ETDRS letters	89 (3)	91 (4)
Spherical equivalent refractive error, median (IQR)	0.0 (0.6)	−0.125 (0.5)
Myopia ≤ − 0.50 D, No. (%)	84 (12)	174 (24)
Incident myopia, No. of cases/No. at risk (%)	–	120/630 (19)
Axial length, mean (SD), mm	23.2 (0.8)	23.5 (0.9)
Five-year change in axial length, median (IQR), μm	–	248 (225)
Subfoveal choroidal thickness, mean (SD), μm	361 (77)	–
Body height, mean (SD), cm	152 (7)	173 (9)
Body weight, median (IQR), kg	40.9 (11)	63.4 (14)
Body mass index, median (IQR), kg/m^2^	17.6 (3)	20.9 (4)

*Abbreviations*: *IQR* interquartile range, *SD* Standard deviation, *ETDRS* Early treatment diabetic retinopathy study

Eyes with myopia at baseline (*n* = 84) increased more in axial length during the 5-year follow-up than eyes without myopia at baseline (454 ± 549 μm vs 243 ± 202 μm, *p* < 0.0001). The effects of both baseline choroidal thickness and baseline axial length on the 5-year change in axial length differed between participants with and without baseline myopia (*p*-values for interaction < 0.0001) and therefore the association-analyses are presented stratified on the presence of myopia at baseline.

The median (IQR) spherical equivalent refraction was − 0.75 (0.50) D in myopic eyes and 0.00 (0.63) D in nonmyopic eyes (*p* < 0.0001; Kruskal-Wallis test). We found no differences in baseline age (11.4 years vs 11.5 years, *p* = 0.067; Kruskal-Wallis Test) or sex distribution (49% vs 44% boys, *p* = 0.39; chi-squared test) between the myopic and nonmyopic participants.

The Pearson correlation coefficient between subfoveal choroidal thickness and axial length at age 11 was − 0.25 in nonmyopic eyes and − 0.53 in myopic eyes.

We tested all associations for interaction with sex both before and after stratifying on the presence of myopia. There were no significant interactions and hence the effect of baseline parameters on the 5-year change in axial length did not differ between sexes (all *p*-values for interaction > 0.53).

### Five-year change in axial length in eyes without myopia at baseline

In the 630 children without myopia at baseline, the 5-year increase in axial length was 34 μm (95%CI 3 to 65, *p* = 0.032; Table 2) higher among girls compared with boys and the difference remained significant after adjusting for baseline axial length, choroidal thickness, body height and age (*p* = 0.0001, Table 2).

**Table 2:** Effect of baseline parameters on the five-year axial length change (μm) from age 11 to 16 years in univariate and multivariate linear regression analyses. Stratified on the presence of baseline myopia

Baseline parameter	Five-year change in axial length (95% CI), crude	*P value*	Five-year change in axial length (95% CI), multivariate*	*P value*
***Eyes without myopia at baseline (n = 630)***				
Subfoveal choroidal thickness, 100 μm	19 (−2 to 39) μm/100 μm	0.071	32 (12 to 53) μm/100 μm	0.0023
Axial length, mm	28 (7 to 49) μm/mm	0.0085	58 (35 to 81) μm/mm	< 0.0001
Body height, cm	−3 (−5 to −1) μm/cm	0.013	−3 (− 5 to − 1) μm/cm	0.0041
Sex (girls vs boys), μm	34 (3 to 65) μm	0.032	64 (31 to 96) μm	0.0001
***Eyes with myopia at baseline (n = 84)***				
Subfoveal choroidal thickness, 100 μm	−167 (−261 to − 74) μm/100 μm	0.0006	−30 (− 129 to 69) μm/100 μm	0.55
Axial length, mm	196 (127 to 265) μm/mm	< 0.0001	216 (129 to 302) μm/mm	< 0.0001
Body height, cm	−5 (−15 to 5) μm/cm	0.35	−10 (− 19 to −0.3) μm/cm	0.044
Sex (girls vs boys), μm	−32 (− 198 to 134) μm	0.70	107 (− 37 to 251) μm	0.14

* Multivariate analysis included: subfoveal choroidal thickness, axial length, body height, sex and age at baseline

Baseline choroidal thickness was not significantly associated with the 5-year change in axial length in the crude analysis (*p* = 0.07, Table 2) but when adjusted for baseline axial length, a thicker choroid associated with greater axial elongation (β = 27 μm/100 μm, 95%CI 6 to 48, *p* = 0.011; data not tabulated) which remained significant when further adjusting for baseline body height, baseline age and sex, (*p* = 0.0023, Table 2).

Five-year increase in axial length was higher the longer the baseline axial length, both in the crude analysis (β = 28 μm/mm, 95%CI 7 to 49, *p* = 0.0085) and when adjusted for baseline choroidal thickness, baseline body height, age at baseline and sex (β = 58 μm/mm, 95%CI 35 to 81, *p* < 0.0001; Table 2).

Axial length increased less during the 5-year study period in children that were taller at baseline (β = − 3 μm/cm, 95%CI − 5 to − 1, *p* = 0.013). This remained significant when adjusting for baseline choroidal thickness, baseline axial length, sex and age at baseline (*p* = 0.0041, Table 2).

### Five-year change in axial length in eyes with myopia at baseline

In the 84 children with myopia at baseline, there was no difference in 5-year change in axial length between girls and boys (*p* = 0.70, Table 2). A thicker choroid at baseline was associated with reduced axial eye elongation in the crude analysis (β = − 167 μm/100 μm, 95%CI -261 to − 74, *p* = 0.0006; Table 2) but not when adjusted for baseline axial length (*p* = 0.34, data not tabulated) or when further adjusting for baseline body height, baseline age and sex (*p* = 0.55, Table 2).

A longer axial length at baseline was associated with greater subsequent 5-year axial elongation (β = 196 μm/mm, 95%CI 127 to 265, *p* < 0.0001; Table 2), an effect that increased when adjusted for baseline choroidal thickness, baseline body height, sex and age at baseline (β = 216 μm/mm, 95%CI 129 to 302, p < 0.0001; Table 2).

Being taller at baseline had no effect on the 5-year change in axial length in the crude analysis (*p* = 0.35) but was associated with less axial elongation when adjusting the analysis for baseline choroidal thickness, baseline axial length, sex and age at baseline (β = − 10 μm/cm, 95%CI − 19 to − 0.3, *p* = 0.044; Table 2).

### Incident myopia

One hundred and twenty (19%) of the 630 children without myopia at baseline developed myopia in their right eyes between age 11 and 16 years. The odds were higher for the girls compared with the boys (OR = 1.72, 95%CI 1.14 to 2.62, *p* = 0.011; Table 3) and the difference remained significant when adjusting for age at baseline and baseline choroidal thickness, axial length and body height (*p* < 0.0001, Table 3).

**Table 3:** Odds ratio for incident myopia at age 16 years in 630 children without myopia at baseline

11-year parameter	Odds Ratio (95% CI) Crude analysis	*P* value	Odds Ratio (95% CI) Multivariate analysis*	*P* value
Subfoveal choroidal thickness, 100 μm	0.80 (0.61 to 1.1)	0.11	0.87 (0.65 to 1.17)	0.37
Axial length, mm	1.57 (1.18 to 2.09)	0.0020	2.10 (1.49 to 2.94)	< 0.0001
Body height, cm	1.00 (0.97 to 1.02)	0.73	0.99 (0.96 to 1.02)	0.37
Sex (girls vs boys)	1.72 (1.14 to 2.62)	0.011	2.68 (1.67 to 4.29)	< 0.0001

* Multivariate analysis included: subfoveal choroidal thickness, axial length, body height, sex and age at baseline

The OR for incident myopia increased by 1.57 (95%CI 1.18 to 2.09, *p* = 0.0020; Table 3) per mm longer eye at baseline, which remained significant when adjusting for baseline choroidal thickness, baseline body height, sex and age at baseline (*p* < 0.0001, Table 3).

Baseline choroidal thickness and body height were not associated with incident myopia (Table 3).

## Discussion

This prospective cohort study showed that a thin choroid at age 11 years did not predict the subsequent five-year axial length elongation nor did it increase the risk of incident myopia. When accounting for axial length, a thicker choroid at 11 years associated with increased eye elongation in nonmyopic eyes. A longer eye at age 11 and female sex associated with an increased five-year elongation in axial length and an increased risk of incident myopia.

The median axial length growth rate in nonmyopic eyes found in present study is comparable to a study of 41 myopic and 60 nonmyopic children aged 10 to 15 years from Australia who were followed over a period of 18-months [18]. Yet, the growth rate among myopic children was somewhat higher in their study compared with ours. Probably caused by their myopic children having a markedly more myopic mean spherical equivalent refraction [23] than the myopic children in present study. A recent three-year longitudinal study of 2408 six-year-old Dutch children found markedly higher axial length growth rates [24] which may be explained by the fact that the Dutch children were 5 years younger at baseline than the Danish children and axial eye growth speed decreases with increasing age [24–27].

In the present study, axial eye elongation increased with baseline choroidal thickness in nonmyopic eyes, whereas baseline choroidal thickness had no effect on axial length change in myopic eyes. Neither was there any effect of baseline choroidal thickness on the incidence of myopia. These findings suggest that a thin choroid does not alter the eye’s susceptibility to axial elongation and myopia development. This is consistent with studies of chicks with experimentally induced myopia where baseline choroidal thickness was found not to predict neither form-deprivation myopia [28, 29] nor myopia induced by hyperopic defocus [29]. Also, it is in line with a recent one-year longitudinal study of 118 Chinese children aged 7–12 years [30] that found choroidal thinning in concert with a myopic shift in refraction, but with no difference in baseline choroidal thickness between eyes with and without a myopic shift. The observation that baseline-nonmyopic eyes elongated more in axial length with increasing baseline choroidal thickness mainly serve to stress these findings. However, it also suggests a link between choroidal thickness and axial eye growth, which seems to disappear on the route to myopia. As this relationship between a thick baseline choroid and greater axial elongation has not been described before, additional studies are needed to confirm the association.

Longer baseline axial length was a predictor for larger subsequent five-year eye length growth in both myopic and nonmyopic eyes. Additionally, the odds of incident myopia increased with increasing baseline axial length. This suggested that children who developed myopia between age 11 and 16 years already were on a myopic track at the age of 11 years. This is consistent with a study from the US of 605 children aged 6 to 14 years [31] where eyes that eventually became myopic were longer than eyes that remained emmetropic. Consequently, children with long eyes at an early age could be a target for preventive myopia strategies such as increased time spent outdoors [32].

Taller body height at age 11 years was associated with reduced elongation of the eye. As body height was not predictive of incident myopia, this association is likely caused by the gradually decrease of axial eye growth with increasing age, and thus with body development.

Axial length increased more in girls than boys and the girls had a higher risk for incident myopia among the children without myopia at baseline. One could speculate that the general pubertal growth spurt also affects the eye. In general, girls enter puberty earlier than boys and the difference in axial eye elongation and incident myopia between sexes may thus be caused by a slightly later pubertal onset and thereby pubertal growth spurt among the boys.

The strengths of the study include its prospective, population-based cohort design and the large number of participants. Limitations include the absence of cycloplegic refraction data. Subjective refraction with fogging can only to some extend substitute cycloplegia [33–36] and it carries the risk of overestimating the prevalence and magnitude of myopia. Consequently, associations with axial length may be more reliable than associations with myopia. The relatively low number of participants with myopia further limits the power to detect associations in this subgroup of participants. No information regarding participant’s ethnicity were available. However, the cohort was found to be representative of the Danish population [37]. In 2017, the Danish population consisted of 69,260 17-years-olds of which 90% were white Caucasians who originates from Denmark (88%) or other Western Countries (2%). Five percentage originated from the Middle East and only 0.1% originated from China and less than 0.1% from other East Asian countries (Statistics Denmark). Another limitation is the limited follow-up rate (52%) and the fact that we only have data from two time points during the follow-up. Short term effects of choroidal thickness on axial elongation were thereby not observable.

## Conclusion

Having a thinner choroid at age 11 years neither increased the subsequent axial eye elongation nor predicted incident myopia.

Children with longer eyes at age 11 experienced greater five-year axial elongation and a markedly higher risk of incident myopia at age 16 years.

## Data Availability

The data that support the findings of this study are available from Statistics Denmark but restrictions apply to the availability of these data, which were used under license for the current study, and so are not publicly available. Data are however available from the authors upon reasonable request and with permission of Statistics Denmark in accordance with Danish regulations and laws.

## Abbreviations

BCVA: Best corrected visual acuity
CI: Confidence interval
EDI-SD-OCT: Enhanced depth imaging spectral domain optical coherence tomography
ETDRS: Early Treatment Diabetic Retinopathy Study
IQR: Interquartile range
OCT: Optical coherence tomography
OR: Odds ratio
SD: Standard deviation
